# Genetic variability of blood groups in southern Brazil

**DOI:** 10.1590/1678-4685-GMB-2018-0327

**Published:** 2020-05-29

**Authors:** Gabriela Waskow, Mirelen Moura de Oliveira Rodrigues, Gabriela Höher, Tor Onsten, Juliana Dal-Ri Lindenau, Marilu Fiegenbaum, Silvana Almeida

**Affiliations:** 1 Universidade Federal de Ciências da Saúde de Porto Alegre (UFCSPA), Programa de Pós-Graduação em Biociências, Porto Alegre, RS, Brazil.; 2 Hospital de Clínicas de Porto Alegre (HCPA), Porto Alegre, RS, Brazil.; 3 Universidade Federal de Santa Catarina (UFSC), Departamento de Biologia Celular, Embriologia e Genética, Florianópolis, SC, Brazil.; 4 Universidade Federal de Ciências da Saúde de Porto Alegre (UFCSPA), Departamento de Ciências Básicas da Saúde, Porto Alegre, RS, Brazil.

**Keywords:** Genotyping techniques, blood group antigens, genetic variation, transfusion reaction, genetic polymorphism

## Abstract

We evaluated genetic variability among the blood groups Kell (*c.578C* > *T* and *c.1790T* > *C*), Kidd (*c.838A* > *G*), Duffy (*c.125A* > *G, c.265C* > *T* and *c.1-67T* > *C*), Diego (*c.2561C* > *T*), MNS (*c.143T* > *C*) and Rh (*c.676G* > *C*) in Rio Grande do Sul in southern Brazil. Genetic profiling from 382 volunteer blood donors was performed through allelic discrimination assays using a hydrolysis probe (TaqMan®) with a real-time PCR system. The sample was divided into two groups: Euro-Brazilian and Afro-Brazilian. A comparison with studies from other regions of Brazil and the 1000 Genomes Database showed significant differences for almost all polymorphisms evaluated in our population. Population differentiation between the Euro- and Afro-Brazilian groups was low (*F*_ST_ value 0.055). However, when each locus was evaluated individually, KEL*06 and FY*02N.01 allele frequencies were significantly higher in the Afro-Brazilian group than in the Euro-Brazilian group. Ethnic classification that uses phenotypic criteria to find blood units with rare antigens may be important when there is a need to detect blood units with an absence of Duffy antigens. There is also a greater probability of finding donors in the Afro-Brazilian group. Taken together, the data indicate strong European and African contributions to the gene pool, with intense admixture.

## Introduction

Blood group systems are characterized by the presence of antigens on red blood cells (RBCs) (Issitt and Anstee, 1998). Currently, 345 blood group antigens are recognized, of which 316 are dispersed among 36 blood group systems (International Society of Blood Transfusion, 2017). Some of these antigens are highly immunogenic, resulting in alloimmunization, hemolytic transfusion reaction (HTR) and hemolytic disease of the fetus and newborn (HDFN) (Anstee, 2009). The main complication directly associated with blood transfusion is HTR, which is mainly induced by the presence of antibodies for blood group antigens (Issitt and Anstee, 1998).

Overall, better characterization of the profiles of blood donors/recipients might increase compatibility and consequently blood transfusion safety. Some rare blood antigens have highly variable frequencies among distinct populations (Pellegrino *et al.*, 2001), which directly impacts transfusion practice, and knowledge of the frequencies of antigens of the main blood groups in each population may help in the search for compatible donors. This is notably true for admixed populations, such as those in Brazil.

Identification of variability in blood groups provides insight into gene ethnic diversity (Pellegrino *et al.*, 2001). Brazil has a territory of continental size, with an area of more than 8 million km^2^ (Instituto Brasileiro de Geografia e Estatística, 2017). Moreover, in Brazil, interethnic crosses from four continents, Europe, Africa, America and Asia, have formed one of the most heterogeneous populations in the world (Parra *et al.*, 2003; Pena *et al.*, 2011; Durso *et al.*, 2014). Although there are data concerning RBC allelic variability throughout the country (Ribeiro *et al.*, 2009; Baleotti *et al.*, 2011; Credidio *et al.*, 2011; Guelsin *et al.*, 2011; Cruz *et al.*, 2012; Faria *et al.*, 2012; Mota *et al.*, 2012; Arnoni *et al.*, 2013; Piassi *et al.*, 2013; Costa *et al.*, 2016a, 2016b; Zacarias *et al.*, 2016), such data are not available for Rio Grande do Sul in southern Brazil. In general, these data may improve the searchability and availability of compatible blood units for patients with antibodies to blood group antigens.

Therefore, the aim of this study was to determine the allelic frequencies of polymorphisms in genes in clinically important blood groups, including Kell (*c.578C* > *T* and *c.1790T* > *C*), Kidd (*c.838A* > *G*), Duffy (*c.125A* > *G, c.265C* > *T* and *c.1-67T* > *C*), Diego (*c.2561C* > *T*), MNS (*c.143T* > *C*), and Rh (*c.676G* > *C*), in blood donors from a city in southern Brazil. Additionally, the study aimed to evaluate whether ethnic classification might improve the search for rare blood units in a blood center.

## Material and Methods

### Sample characterization

A total sample of 382 regular repetitive voluntary blood donors of both sexes was collected from the Blood Bank of Hospital de Clínicas de Porto Alegre (HCPA), Rio Grande do Sul, Brazil (30°01’59”S 51°13’48”), between 2012 and 2015. The population from southern Brazil is ethnically admixed (Santos, 2002; Flôres *et al.*, 2014); thus, the sample was divided into Euro-Brazilian (n= 334) and Afro-Brazilian (n= 48) groups. This classification was performed by trained blood bank professionals according to the following phenotypic characteristics: color and texture of hair, skin color in the medial part of the arm, and the shape of the nose and lips (Parra *et al.*, 2003). This is a standard classification used to assist rare phenotypes.

The blood donors agreed to participate through written informed consent. This study was approved by the Ethics Research Committee of the Hospital de Clínicas de Porto Alegre (No: 110418) and the Universidade Federal de Ciências da Saúde de Porto Alegre (UFCSPA) (No: 1829-12).

### DNA extraction and genotyping

Genomic DNA was extracted from peripheral blood leukocytes by a standard salting out procedure (Lahiri and Nurnberger, 1991). The DNA samples were quantified based on optical density at 260 nm (BioSpec-Nano, Shimadzu, Columbia, MD) and diluted to 10 ng/μL.

As listed in Table 1, single nucleotide polymorphisms (SNPs) *c.578C* > *T* and *c.1790T* > *C* in *KEL* (Kell, rs8176058 and rs8176038), *c.838A* > *G* in *SLC14A1* (Kidd, rs1058396), *c.125A* > *G, c.265C* > *T* and *c.1-67T* > *C* in *ACKR1* (Duffy, rs12075, rs34599082 and rs2814778), *c.2561C* > *T* in *SLC4A1* (Diego, rs2285644), *c.143T* > *C* in *GYPB* (MNS, rs7683365), and c.676G > C in *RHCE* (Rh, rs609320) were analyzed by allelic discrimination using TaqMan 5’-nuclease assays with a real-time PCR system (StepOnePlus, Applied Biosystems, Foster City, CA, USA). The following assays were used: AH8979I, C_25596899_20, C_1727582_10, C_2493442_10, C_11324554_10, C_15769614_10, C_26654865_10, C_34183121_10, and AH5I4HL (ThermoFisher Scientific, Waltham, MA). The reactions were performed with fast thermal cycling conditions with 1X TaqMan® genotyping master mix, 1X TaqMan® genotyping assay, 10 ng of DNA and nuclease-free water (final volume 8 μL).

**Table 1:** Characterization of blood groups alleles evaluated in present study.

BGS	Gene	Allele	dbSNP	Nucleotide	Amino acid
Kell	*KEL*	KEL*01	rs8176058	c.578C > T	p.Thr193Met
		KEL*02			
Kell	*KEL*	KEL*06	rs8176038	c.1790T > C	p.Leu597Pro
		KEL*07			
Kidd	*SLC14A1*	JK*01	rs1058396	c.838A > G	p.Asn280Asp
		JK*02			
Duffy	*ACKR1*	FY*01	rs12075	c.125A > G	p.Asp42Gly
		FY*02			
Duffy	*ACKR1*	FY*02W.01	rs34599082	c.265C > T	p.Arg89Cys
Duffy	*ACKR1*	FY*02N.01	rs2814778	c.1-67T > C	p.0
Diego	*SLC4A1*	DI*01	rs2285644	c.2561C > T	p.Pro854Leu
		DI*02			
MNS	*GYPB*	GYPB*S	rs7683365	c.143T > C	p.Met48Thr
		GYPB*s			
Rh	*RHCE*	RHCE*E	rs609320	c.676G > C	p.Ala226Pro
		RHCE*e			

Data obtained from ISBT; (Issitt and Crookston, 1984).

BGS = Blood group system.

### Statistical analyses

A chi-square adjustment test was applied to determine whether the distribution of observed genotype frequencies agreed with those expected under Hardy-Weinberg equilibrium (HWE). We compared the allele frequencies in the present study with data in the 1000 Genomes database (Ensembl GRCh38 – phase III) (African – AFR, European – EUR, East Asian – EAS, South Asian – SAS, and Admixed American - AMR) and data for blood donors from other states of Brazil [Santa Catarina - SC (Costa *et al.*, 2016a,b), Paraná – PR-POP1 and PR-POP2 (Guelsin *et al.*, 2011; Zacarias *et al.*, 2016), São Paulo - SP (Ribeiro *et al.*, 2009), Bahia – BA (Costa *et al.*, 2016a,b), and Minas Gerais – MG (Alves *et al.*, 2018)]. Comparison of allelic frequencies was performed using Fisher's exact test with R software in the Rcmdr package (Fox, 2005). A *p*-value < 0.05 was considered significant.

Genetic distance was determined as *F*_ST_ using the Arlequin v.3.5 program (Excoffier and Lischer, 2010), and 95% confidence intervals were estimated with R software using the diveRsity package with 3000 bootstraps (Keenan and McGinnity, 2013).

## Results

The distribution of genotype frequencies was in HWE. Minor allele frequencies (MAFs) of the investigated polymorphisms in the Euro- and Afro-Brazilians of our sample, 1000 Genomes database, and data of blood donors from other states of Brazil are shown in Table 2. In our study, KEL*06 and FY*02N.01 allele frequencies differed between the Euro- and Afro-Brazilian subgroups (*p*=0.023 and *p* < 0.001, respectively; Table 2). When compared to the 1000 Genomes database, the allele frequencies of our Euro-Brazilians were different from those described for AFR, EAS, and SAS populations, except for the GYPB*S allele in the SAS population and the KEL*06 allele in the EAS and SAS populations. When compared to EUR, the allele frequencies in Euro-Brazilians for JK*02 (*p*=0.002), FY*02N.01 (*p* < 0.001,) and DI*01 (*p*=0.002) variants differed. Comparison with the AMR population also revealed differences in JK*02 (*p* < 0.001), DI*01 (*p* < 0.001), and RHCE*E (*p* < 0.001) variants. Comparison of Euro-Brazilians with blood donors from other regions of Brazil indicated differences in allele frequencies for FY*01 (*p* < 0.001), FY*02N.01 (*p*=0.047), and DI*01 (*p*=0.014) from SC; JK*02 (*p*=0.013), FY*02N.01 (*p* < 0.001), and RHCE*E (*p*=0.048) from PR-POP1; JK*02 (p=0.016), FY*01 (p=0.003), and FY*02N.01 (*p*=0.004) from PR-POP2; FY*01 (*p* < 0.001), FY*02N.01 (*p* < 0.001), and GYPB*S (*p*=0.047) from SP; and FY*01 (*p*=0.005 and *p* < 0.001) and FY*02N.01 (*p* < 0.001 and *p* < 0.001) from BA and MG, respectively.

**Table 2:** Minor allele frequencies of blood groups variants in Euro and Afro-Brazilians from Rio Grande do Sul, 1000 Genomes Database and previous studies performed at Brazil.

Sample (n)	KEL*01	KEL*06	JK*02	FY*01	FY*02W.01	FY*02N.01	DI*01	GYPB*S	RHCE*E
Euro-Brazilians (334)	0.033	0.003	0.424	0.444	0.015	0.076	0.010	0.320	0.154
Afro-Brazilians (48)	0.010	0.021	0.353	0.357	0.000	0.408	0.011	0.350	0.166
*p*	*ns*	*0.023*	*ns*	*ns*	*ns*	*< 0.001*	*ns*	*ns*	*ns*
EUR (503)^1^	0.038	0.000§	0.501¥ §	0.398	0.013	0.006¥ §	0.000¥	0.339	0.160
AFR (661)^2^	0.002¥	0.099¥ §	0.228¥ §	0.019¥ §	0.000¥ £	0.964¥ §	0.001¥	0.185¥ §	0.080¥
EAS (504)^3^	0.000¥	0.000§	0.526¥ §	0.923¥ §	0.001¥	0.000¥ §	0.026¥	0.035¥ §	0.202¥
SAS (489)^4^	0.006¥	0.000§	0.371¥	0.640¥ §	0.004¥	0.000¥ §	0.002¥	0.325	0.090¥ §
AMR (347)^5^	0.022	0.009	0.519¥ §	0.461	0.007	0.078§	0.052¥	0.344	0.232¥
SC (373)^6^	0.030	nt	0.460	0.560¥ §	nt	0.050¥ §	0.030¥	nt	0.150
PR-POP1 (251)^7^	0.043	nt	0.498¥ §	0.436	nt	0.023¥ §	0.022	nt	0.113¥
PR-POP2 (400)^8^	0.028	nt	0.488¥ §	0.365¥	nt	0.123¥ §	nt	nt	0.151
SP (948)^9^	0.024	nt	0.460§	0.360¥	0.016	0.185¥ §	0.020	0.279¥	0.150
BA (196)^10^	0.020	nt	0.380	0.357¥	nt	0.436¥	0.020	nt	0.110
MG (170)^11^	0.015	nt	0.406	0.297¥	nt	0.229¥ §	nt	nt	0.126

Data are presented as relative frequency. Euro-Brazilians and Afro-Brazilian, present study. ns, non-significant; nt, non-tested;

¥ *p*-value < 0.05 when compared with Euro-Brazilians;

§ *p*-value < 0.05 when compared with Afro-Brazilians;

£ statistical analysis for comparison between Afro-Brazilians and AFR cannot be performed.

1 EUR: European (CEU, Utah Residents (CEPH) with Northern and Western Ancestry; TSI, Toscani in Italia; FIN, Finnish in Finland; GBR, British in England and Scotland; IBS, Iberian Population in Spain).

2 AFR: African (YRI, Yoruba in Ibadan, Nigeria; LWK, Luhya in Webuye, Kenya; GWD, Gambian in Western Divisions in the Gambia; MSL, Mende in Sierra Leone; ESN, Esan in Nigeria; ASW, Americans of African Ancestry in the SW USA; ACB, African Caribbeans in Barbados).

3 EAS: East Asian (CHB, Han Chinese in Beijing, China; JPT, Japanese in Tokyo, Japan; CHS, Southern Han Chinese; CDX, Chinese Dai in Xishuangbanna, China; KHV, Kinh in Ho Chi Minh City, Vietnam).

4 SAS: South Asian (IH, Gujarati Indians from Houston, Texas; PJL, Punjabi from Lahore, Pakistan; BEB, Bengali from Bangladesh; STU, Sri Lankan Tamils from the UK; ITU, Indian Telugu from the UK).

5 AMR: Admixed American (MXL, Mexican Ancestry from Los Angeles USA; PUR, Puerto Ricans from Puerto Rico; CLM, Colombians from Medellin, Colombia; PEL, Peruvians from Lima, Peru) of 1000 Genomes Project;

6 SC: Blood donors from the state of Santa Catarina (Costa *et al.* (2016a and 2016b);

7 PR-POP1: Blood donors from the Southwest region of the state of Parana (Zacarias *et al.* (2016),

8 PR-POP2: Blood donors from the state of Parana (Guelsin *et al.* (2011),

9 SP: Blood donors from the state of São Paulo (Ribeiro *et al.*, 2009),

10 BA: Admixed population from the state of Bahia (Costa *et al.*, 2016a and 2016b and

11 MG: Blood donors from the state of Minas Gerais (Alves *et al.*, 2018).

The allele frequencies for KEL*06 observed in Afro-Brazilians were similar only in AMR (Table 2), though the JK*02 allele distribution in Afro-Brazilians was similar to that observed in the SAS, SC, BA and MG populations. Moreover, the FY*01 allele frequency in Afro-Brazilians was similar to that in the EUR, AMR, PR, SP, BA and MG populations, but the FY*02N.01 allele frequency observed in the present sample was different in all populations, except for BA (Table 2). The frequency of the DI*01 variant was similar in all populations assessed. The GYPB*S variant showed different distributions in AFR (*p* < 0.001) and EAS (*p* < 0.001) and the RHCE*E variant in SAS (*p*=0.028).

**Figure 1:**
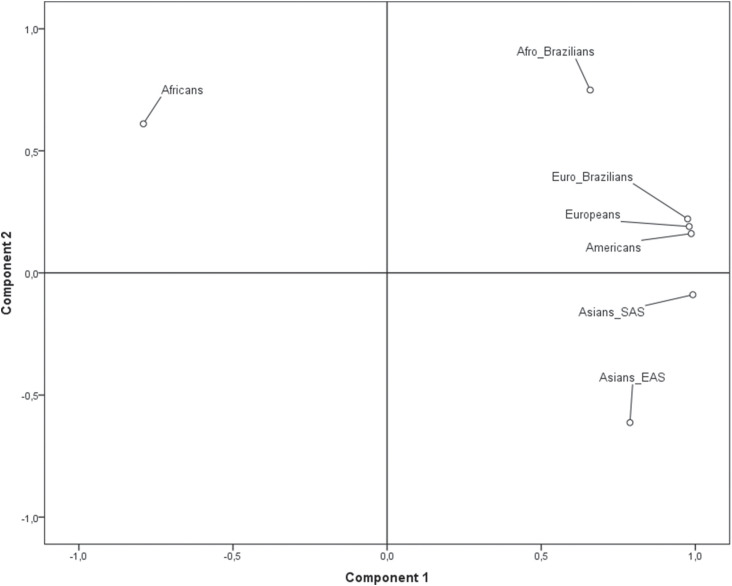
Genetic distance among the populations analyzed in this study based on blood group alleles.

Pairwise *F*_ST_ values for the samples of the present study and the 1000 Genomes database are shown in Table 3. Low genetic distance (*F*_ST_=0.055) between Euro- and Afro-Brazilians of Rio Grande do Sul was observed when evaluated for population differentiation. In relation to EUR populations, the Euro- and Afro-Brazilian groups showed the lowest *F*_ST_ values (0.004 and 0.080, respectively), and lower *F*_ST_ values were also observed in comparisons of our groups and AMR populations (0.009 for Euro- and 0.061 for Afro-Brazilians). In contrast, the highest genetic distance was found between Euro-Brazilians of Rio Grande do Sul and the AFR population (0.431) and between Afro-Brazilians and AFR (0.297). Figure 1 shows the genetic distance observed for the populations analyzed in this study based on blood group alleles. The main result of *F*_ST_ analysis indicated that the AFR population is genetically more distinct than the other populations. To evaluate the contribution of each variant to the genetic distance observed, *F*_*ST*_ values were also estimated for each SNP by examining the present sample and the 1000 Genomes Database. According to *F*_ST_ values, rs2814778 (*c.1-67T* > *C*) and rs12075 (*c.125A* > *G*) polymorphisms in the *ACKR1* gene (Duffy blood group) present high differentiation among populations (0.865 and 0.399, respectively, Table 4).

**Table 3:** Pairwise *F*_*ST*_ among South Brazilian population and populations evaluated in 1000 Genome Database.

	Euro-descendants	Afro-descendants	AFR^1^	EUR^2^	EAS^3^	SAS^4^	AMR^5^
Euro-descendants	–						
Afro-descendants	0.055 (0.021 – 0.097)	–					
AFR^1^	0.431 (0.410 – 0.450)	0.297 (0.218 – 0.370)	–				
EUR^2^	0.004 (0.001 – 0.008)	0.080 (0.042 – 0.131)	0.456 (0.441 – 0.469)	–			
EAS^3^	0.200 (0.178 – 0.222)	0.350 (0.274 – 0.415)	0.633 (0.621 – 0.646)	0.205 (0.186 – 0.224)	–		
SAS^4^	0.037 (0.024 – 0.051)	0.137 (0.080 – 0.199)	0.514 (0.499 – 0.529)	0.046 (0.034 – 0.060)	0.132 (0.116 – 0.150)	–	
AMR^5^	0.009 (0.003 – 0.016)	0.061 (0.024 – 0.104)	0.438 (0.417 – 0.459)	0.009 (0.005 – 0.015)	0.174 (0.154 – 0.195)	0.044 (0.033 – 0.058)	–

Data are presented as *F*_ST_ (95% confidence interval).

1 AFR: African (YRI, Yoruba in Ibadan, Nigeria; LWK, Luhya in Webuye, Kenya; GWD, Gambian in Western Divisions in the Gambia; MSL, Mende in Sierra Leone; ESN, Esan in Nigeria; ASW, Americans of African Ancestry in the SW USA; ACB, African Caribbeans in Barbados).

2 EUR: European (CEU, Utah Residents (CEPH) with Northern and Western Ancestry; TSI, Toscani in Italia; FIN, Finnish in Finland; GBR, British in England and Scotland; IBS, Iberian Population in Spain).

3 EAS: East Asian (CHB, Han Chinese in Beijing, China; JPT, Japanese in Tokyo, Japan; CHS, Southern Han Chinese; CDX, Chinese Dai in Xishuangbanna, China; KHV, Kinh in Ho Chi Minh City, Vietnam).

4 SAS: South Asian (IH, Gujarati Indians from Houston, Texas; PJL, Punjabi from Lahore, Pakistan; BEB, Bengali from Bangladesh; STU, Sri Lankan Tamils from the UK; ITU, Indian Telugu from the UK).

5 AMR: Admixed American (MXL, Mexican Ancestry from Los Angeles USA; PUR, Puerto Ricans from Puerto Rico; CLM, Colombians from Medellin, Colombia; PEL, Peruvians from Lima, Peru).

**Table 4:** SNPs population differentiation.

Gene	dbSNP	*F*_*ST*_ (95% CI)
*ACKR1*	rs34599082	0.007 (0.003 – 0.012)
*KEL*	rs8176058	0.018 (0.012 – 0.025)
*SLC4A1*	rs2285644	0.028 (0.018 – 0.040)
*RHCE*	rs609320	0.029 (0.020 – 0.038)
*SLC14A1*	rs1058396	0.064 (0.051 – 0.079)
*KEL*	rs8176038	0.082 (0.066 – 0.097)
*GYPB*	rs7683365	0.083 (0.072 – 0.094)
*ACKR1*	rs12075	0.399 (0.384 – 0.413)
*ACKR1*	rs2814778	0.865 (0.847 – 0.881)

## Discussion

We examined population differentiation for the distribution of blood group alleles in blood donors from Rio Grande do Sul. Analysis of all variants together demonstrated that Euro- and Afro-Brazilian individuals from Rio Grande do Sul are genetically close (*F*_*ST*_=0.055; Table 3). However, when each locus was evaluated individually, KEL*06 and FY*02N.01 allele frequencies were found to be significantly higher in the Afro-Brazilian group when compared with the Euro-Brazilian group (Table 2). Furthermore, based on data for AFR and EUR populations of the 1000 Genomes database, KEL*06 allele frequencies differ between these continents, with higher frequencies in AFR than in EUR. The Fy^a^ and Fy^b^ antigens of the Duffy blood group act as receptors for malarial parasites on human RBCs (Miller *et al.*, 1976). The FY*02N.01 allele predominates in malaria-endemic areas, such as some Africa regions, because it prevents expression of the receptor on the erythrocyte membrane, and consequently, these erythrocytes become refractory to infection by malarial parasites. These antigenic determinants have been used for determining ethnic composition and as anthropological markers (Cavasini *et al.*, 2007), possibly due to their impact on natural selection in different geographical regions (Miller *et al.*, 1976; Hamblin *et al.*, 2002). Taken together, these findings for KEL*06 and FY*02N.01 allele frequencies distributions indicate, as expected, a greater African background in the genetic pool of Afro-Brazilians than in Euro-Brazilians in Rio Grande do Sul.

Based on the comparison of allele frequencies, which was conducted separately for blood group systems, it is evident that our Euro-Brazilian group had more similarities with the EUR and AMR populations in the 1000 Genomes database than with the AFR, EAS and SAS populations (Table 2). Although some significant differences have been reported, the frequencies observed for these alleles were not highly discrepant among these populations. Moreover, these findings were corroborated by analyses of all genetic markers together (EUR: *F*_*ST*_=0.004 and AMR: *F*_*ST*_=0.009, Table 3). In the same way, the Afro-Brazilians were found to be closer to EUR (*F*_*ST*_=0.080) and AMR (*F*_*ST*_=0.061) than to AFR (*F*_*ST*_=0.297) populations. It is important to emphasize that the classification of ancestry in our sample was performed according to phenotypic characteristics, as it is performed in the blood bank. Previous studies have also demonstrated a discrepancy between skin color information and genetic ancestry (Boquett *et al.*, 2015; Lima-Costa *et al.*, 2015). For example, Boquett *et al.* (2015) evaluated self-assessed skin color and HLA genetic information of bone marrow donors from the state of Rio Grande do Sul and found that Brazilian individuals self-assessing as Black were closer genetically to European populations than to African populations (Boquett *et al.*, 2015). Lima-Costa *et al.* (2015) also demonstrated that the association between ethnoracial self-classification and genome-based ancestry is not linear.

Although the KEL*06 and FY*02N.01 allele frequencies indicated more African ancestry in the Afro-Brazilian group than in the Euro-Brazilian group, these allele frequencies in the former are intermediate between the AFR and EUR populations. These findings were expected due to the colonization process of southern Brazil, which is predominantly characterized by admixture between European descendants. Consequently, this population has a distinct genetic background in relation to populations from other Brazilian regions (Pena *et al.*, 2011; Salzano and Sans, 2014).

When our data were compared to allele frequencies of blood donors from Santa Catarina (Costa *et al.*, 2016a,b), Paraná (Guelsin *et al.*, 2011; Zacarias *et al.*, 2016), São Paulo (Ribeiro *et al.*, 2009), Bahia (Costa *et al.*, 2016a,b) and Minas Gerais (Alves *et al.*, 2018), JK*02, FY*01 and FY*02N.01 variants presented greater differences in frequency among Brazilian regions (Table 2). Regardless of the similarities in the ancestral process of colonization among some localities, European ancestry is uniformly preponderant in southern Brazil. For instance, in the Rio Grande do Sul population, the composition of Europeans, Africans, and Amerindians is 72.9%, 14%, and 13%, respectively. In Santa Catarina, it is 79.7%, 11.4%, and 8.9%, respectively. In the state of Paraná, the average individual has 71% European ancestry, followed by 17.5% African, and 11.5% Amerindian. In São Paulo, the genetic background of the population is composed of 62.9% European, 25.5% African, and 11.6% Amerindian. In Minas Gerais, it is 59.2%, 28.9%, and 11.9%, respectively (Manta *et al.*, 2013). The genomic ancestry of the Bahia population is 42.4% European, 50.5% African, and 5.8% Amerindian (Lima-Costa *et al.*, 2015). Despite observed interethnic genetic similarity, there are significant differences in the frequencies of RBC polymorphisms among these populations. This suggests that data must be well documented and considered within the perspective of transfusion medicine.

Although similarity was demonstrated between Euro- and Afro-Brazilians when all variants were analyzed together, the ethnic classification that uses phenotypic criteria to find blood units with rare antigens may be important when the KEL*06 and, mainly, FY*02N.01 alleles are considered for this southern Brazilian population. Thus, when there is a need to detect blood units with an absence of Duffy antigens, there is a greater probability of finding donors in this group. To the best of our knowledge, no other studies have reported RBC genetic variability in Rio Grande do Sul, emphasizing the intense process of admixture that makes the Brazilian population unique in its ethnic background.
